# Role of chaperone-mediated autophagy in the pathophysiology including pulmonary disorders

**DOI:** 10.1186/s41232-021-00180-9

**Published:** 2021-10-01

**Authors:** Yusuke Hosaka, Jun Araya, Yu Fujita, Kazuyoshi Kuwano

**Affiliations:** Division of Respiratory Diseases, Department of Internal Medicine, The Jikei University School of Medicine, 3-25-8 Nishi-shimbashi, Minato-ku, Tokyo, 105-8461 Japan

**Keywords:** Autophagy, Chaperone-mediated autophagy, Chronic obstructive pulmonary disease, Lung cancer

## Abstract

Autophagy is a highly conserved mechanism of delivering cytoplasmic components for lysosomal degradation. Among the three major autophagic pathways, chaperone-mediated autophagy (CMA) is primarily characterized by its selective nature of protein degradation, which is mediated by heat shock cognate 71 kDa protein (HSC70: also known as HSPA8) recognition of the KFERQ peptide motif in target proteins. Lysosome-associated membrane protein type 2A (LAMP2A) is responsible for substrate binding and internalization to lysosomes, and thus, the lysosomal expression level of LAMP2A is a rate-limiting factor for CMA. Recent advances have uncovered not only physiological but also pathological role of CMA in multiple organs, including neurodegenerative disorders, kidney diseases, liver diseases, heart diseases, and cancers through the accumulation of unwanted proteins or increased degradation of target proteins with concomitant metabolic alterations resulting from CMA malfunction. With respect to pulmonary disorders, the involvement of CMA has been demonstrated in lung cancer and chronic obstructive pulmonary disease (COPD) pathogenesis through regulating apoptosis. Further understanding of CMA machinery may shed light on the molecular mechanisms of refractory disorders and lead to novel treatment modalities through CMA modulation.

## Background

**Fig. 1:**
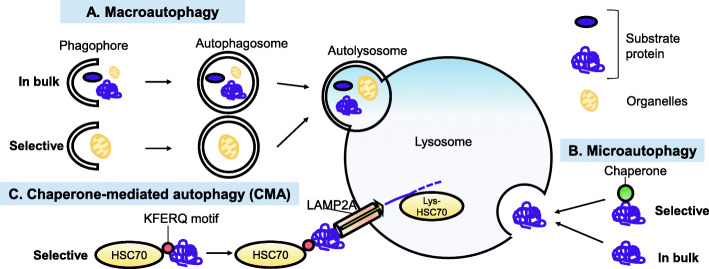
Three forms of autophagy. Cellular components can be delivered into lysosomes for degradation through three distinct autophagic pathways. **A** During the process of macroautophagy, substrate proteins and organelles are sequestered by autophagosome. Fusion of a lysosome with the autophagosome to form the autolysosome is crucial process for degradation. **B** During microautophagy, small components of the cytoplasm are engulfed by direct invagination into lysosomes. **C** CMA degrades soluble proteins containing KFERQ-like motif, which are specifically recognized by chaperone HSC70 and co-chaperones. Lysosome-associated membrane protein type 2A (LAMP2A) is responsible for substrate binding and internalization to lysosome

Autophagy is a highly conserved mechanism of delivering cytoplasmic components for lysosomal degradation to maintain the homeostatic balance between the synthesis, degradation, and recycling of cellular proteins and organelles [1]. Three forms of distinct autophagy have been identified: chaperon-mediated autophagy (CMA), microautophagy, and macroautophagy [1] (Fig. 1). CMA is a type of selective autophagy for the lysosomal degradation of proteins with the KFERQ peptide motif. Microautophagy requires small components of the cytoplasm to be engulfed by direct invagination into lysosomes. During the process of macroautophagy, substrate proteins and organelles are sequestered by the autophagosome. The fusion of a lysosome with the autophagosome to form the autolysosome is a crucial process for degradation [1]. Because macroautophagy is the best-characterized form of autophagy, recent studies of the molecular mechanisms and pathophysiological effects of autophagy have mainly focused on macroautophagy. We have reported pathogenic involvement of macroautophagy in idiopathic pulmonary fibrosis (IPF), a form of progressive fibrosing interstitial pneumonia and in chronic obstructive pulmonary disease (COPD), which is characterized by progressive airflow limitation mainly caused by cigarette smoke (CS) exposure [2, 3]. Both IPF and COPD are aging-associated pulmonary disorders and the lysosomal function declines with aging [4]. Insufficient macroautophagy, including mitochondria-selective mitophagy enhances mitochondrial reactive oxygen species (ROS) production, which regulates cellular senescence in epithelial cells and myofibroblast differentiation in fibroblasts in terms of COPD and IPF pathogenesis [5–7]. It has been recognized that there is crosstalk between macroautophagy and CMA, and they can at least partly compensate for their functional loss each other, indicating that CMA activity may has a crucial role in the pathogenesis of aging-associated pulmonary disorders with insufficient macroautophagy [8]. The essential CMA components have been detected only in birds and mammals; hence, the paucity of appropriate model systems such as yeast and flies may explain the delayed elucidation of CMA [8]. However, the physiological and pathological role of CMA is being increasingly uncovered using developed genetic manipulation technologies. Among the three major autophagic pathways, CMA is primarily characterized by the selective nature of protein degradation, which is mediated by heat shock cognate 71 kDa protein (HSC70: also known as HSPA8) recognition of the KFERQ peptide motif in target proteins [8]. Approximately 40% of proteins in the mammalian proteome contain a canonical KFERQ-like motif; hence, CMA-modulated proteostasis may have an essential physiological role in regulating a wide array of cellular processes, including glucose and fat metabolism, transcription, immune responses, and the cell cycle [8]. Lysosome-associated membrane protein type 2A (LAMP2A) is responsible for substrate binding and internalization in lysosomes. Malfunction of CMA has also been implicated in a variety of pathologies, including neurodegenerative disorders, kidney diseases, liver diseases, heart diseases, and cancers through the accumulation of unwanted proteins or increased degradation of target proteins with concomitant metabolic alterations [8]. With respect to pulmonary disorders, the involvement of CMA has been demonstrated in lung cancer progression and we have recently reported CMA-mediated tumor growth and chemoresistance in lung cancer pathogenesis [9]. COPD is a representative aging-related pulmonary disorder mainly caused by CS exposure, and aberrant macroautophagic activity has been implicated in its pathogenesis [3, 10–12]. We have also shown the potential participation of CMA in COPD through regulating the unfolded protein response (UPR) and apoptosis [13], indicating that CMA may have an important role in a variety of pulmonary pathogenesis. In this review, we initially summarize the general mechanism and role of CMA, and then describe the involvement of CMA in the pathogenesis of lung cancer and COPD.

## Mechanisms of CMA

CMA is responsible for the selective degradation of soluble cytosolic proteins with the KFERQ peptide motif or a related sequence [8]. During the initial step of CMA, HSC70-mediated cytosolic recognition of the KFERQ peptide motif in proteins determines target selectivity. Although approximately 40% of proteins in the mammalian proteome contain a canonical KFERQ-like motif, this targeting motif can be generated through post-translational modifications, such as phosphorylation, resulting in further expansion of potential CMA substrates [14]. Cytosolic HSC70 binds the KFERQ-motif on targeted proteins and co-chaperones, including carboxyl terminus of HSC70-interacting protein (CHIP), heat shock protein 40 (HSP40), HSP70-intercting protein (HIP), and HSP70-HSP90 organizing protein (HOP), which participate in the mechanism for substrate unfolding, and are prerequisite for lysosomal translocation [8]. HSC70 is also present in the lysosomal lumen and is necessary for completing translocation of the substrate, which is also a crucial step for CMA progression [15]. The role of HSC70 in autophagy is not restricted to CMA but is also linked to chaperone-assisted selective autophagy (a type of selective macroautophagy for ubiquitin-positive protein aggregates) [16] and microautophagy [17], suggesting the potential implication of HSC70 in conducting all type of autophagic processes [8].

LAMP2 is a lysosomal component that is indispensable for completing CMA [8]. Among the three isoforms of LAMP2 (LAMP2A, 2B, and 2C), LAMP2A is the only isoform that is necessary for the CMA machinery. LAMP2A is responsible for both substrate binding and internalization to lysosomes. The cytosolic tail domain of LAMP2A is crucially required for binding to substrate complexes containing HSC70 [18]. Multimerization of LAMP2A to form a 700 kDa protein complex, a transmembrane protein channel is an essential process for substrate translocation into the lumen [19]. The lysosomal expression level of LAMP2A is considered to be the rate-limiting factor for CMA activity and can be altered by synthesis, degradation, and redistribution [8]. Oxidative stress and genotoxic damage have been shown to induce de novo synthesis of LAMP2A [20, 21], indicating a protective role for CMA in responding to cell stress conditions. Although the detailed mechanisms for transcriptional upregulation of LAMP2A remain uncertain, the involvement of nuclear factor of activated T cells (NFAT1) has been reported in the transcriptional upregulation of LAMP2A in T cells [22]. Nrf2 is a master transcription factor that orchestrates the antioxidant defense system via expression of a wide array of antioxidant enzymes for redox homeostasis and cell survival in response to oxidative stress [23]. It has been reported that forced Nrf2 expression prevents CMA decline and has a neuroprotective effect in a mouse model of Parkinson’s disease [24]. A recent paper has demonstrated that Nrf2 upregulates LAMP2A expression levels by binding to the *LAMP2A* gene [25], which may at least partly explain the mechanism for CMA activation during oxidative stress. We have also detected the participation of Nrf2-mediated LAMP2A expression in cigarette smoke (CS)-induced CMA activation [13]. Alternatively, increase in LAMP2A levels is also mediated through alterations of protein stability in starvation conditions. An extension of half-life of LAMP2A from 36 h to 72 h has been reported in cultured hepatocytes and fibroblasts in response to prolonged starvation [26].

In addition to basal CMA activity, CMA is upregulated in response to a wide array of stressors, including starvation, oxidative stress, genotoxic stress, hypoxia, and radiation, indicating the presence of fine-tuning mechanisms for CMA [8]. Actually, there are several signaling pathways regulating CMA activity, including the calcineurin-NFAT pathway in CMA activation in T cells, PARα signaling in CMA inhibition, and the TOR complex2 (TORC2)-AKT1-PHLPP1 axis in CMA inhibition [8]. However, involvement of pathway-mediated regulation of CMA activity in both physiological and pathological settings remains uncertain especially in pulmonary disorders.

## Physiological roles of CMA

**Fig. 2:**
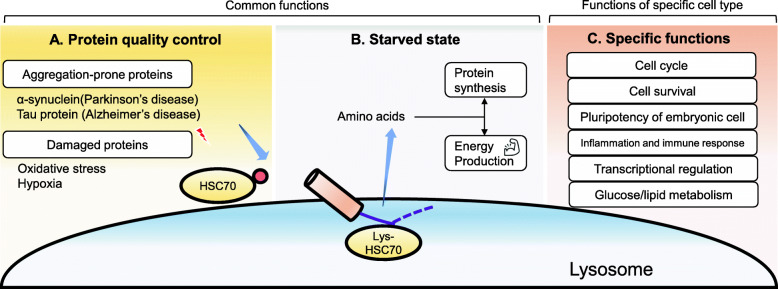
Physiological roles of CMA. CMA has an essential role in physiological processes, including proteostasis, cellular energetics, cell cycle, cell survival, cell stemness, immune responses, and metabolic pathways

CMA substrates with a targeting KFERQ motif include approximately 40% of proteins in the mammalian proteome and potential CMA substrates can be further generated by post-translational modifications; hence, it is not surprising that CMA is responsible for a wide array of physiological processes for maintaining cellular function (Fig. 2). CMA is activated by a variety of stressors and has an essential role in preserving proteostasis. Impairment of CMA activity results in the accumulation of oxidized and aggregated proteins [27]. In the manner similar to macroautophagy, CMA activation is considered to be a part of the first line of defense against stress-induced aggregation of damaged and misfolded proteins to improve cellular resistance to proteotoxicity [8]. Starvation can activate both macroautophagy and CMA, but the timing of activation is different. Initially, macroautophagy is activated and CMA is subsequently upregulated and sustained in condition of prolonged nutrient deprivation. CMA is mainly responsible for replenishment of intracellular amino acids for maintaining protein synthesis and gluconeogenesis. Furthermore, it has been reported that CMA participates in the regulation of glucose and lipid metabolism through timely selective degradation of key enzymes in these pathways in terms of selective proteome remodeling, indicating that CMA may have a dominant role in controlling metabolic pathways and cellular energetics [8]. The majority of glycolytic enzymes are selective targets for CMA degradation, especially in conditions of starvation. Proteins that participate in lipid metabolism, including lipogenesis enzymes and lipid droplet coat proteins, have also been demonstrated to be selective targets for CMA degradation. CMA may maintain intracellular lipid levels by regulating both lipogenesis and lipolysis [8].

CMA is involved in controlling the cell cycle through the degradation of target proteins associated with cell cycle regulation. Checkpoint kinase 1 (CHK1) activation is involved in both normal and DNA damage-induced cell cycle arrest and CHK1 is a CMA substrate [28, 29]. Hypoxia-inducible factor-1 subunit α (HIF-1α) is a CMA substrate and is responsible for cell cycle arrest as an adaptive response to oxygen deprivation [30]. HIF-1α-mediated cell cycle arrest is linked to increased expression levels of the cyclin-dependent kinase (CDK) inhibitors p21 and p27. MYC is a transcription factor and can stimulate cell cycle progression through a variety of mechanisms, including cyclins, CDK, and E2F transcription factors [29, 31]. It has been demonstrated that CMA regulates MYC expression levels via an indirect mechanism [29]. Accordingly, CMA may regulate both cell cycle arrest and progression through the fine-tuning of degradation or accumulation of those target proteins, which is governed by specific stimuli and conditions.

It has been reported that CMA activity is a critical determinant of pluripotency of embryonic stem cells. Controlled low baseline CMA activity promotes self-renewal, but upregulation enhances differentiation through regulating intracellular α-ketoglutarate level, which modulate histone and DNA methylation [32]. Furthermore, a recent study has shown a pivotal role for CMA in maintaining hematopoietic stem-cell (HSC) function through protein quality control and appropriate energetic supply over a lifespan; aging-linked pathological decreases in CMA activity are associated with the functional decline of HSC capacity for self-renewal and multilineage repopulation, which can be restored by genetic or pharmacological activation of CMA [33]. It is likely that both quality control by degrading unwanted oxidized protein and appropriate energetic supply by regulating metabolic enzymes are necessary for CMA-mediated maintenance of cell stemness.

CMA is also involved in the regulation of inflammation and immune responses through a variety of mechanisms. Nuclear factor-κB (NF-κB), a representative proinflammatory transcription factor, can be activated by CMA-mediated degradation of NF-κB inhibitor-α (IκBα) [34]. CMA regulates CD4^+^ T cell activation through selective degradation of negative regulators of T cell response signaling [22]. The cyclic GMP-AMP synthase (cGAS)-STING pathway, which provides an innate immune antiviral response can be controlled by CMA especially in the late phase of infection. Deactivation of the cGAS-STING pathway is induced by CMA-mediated degradation of desumoylated STING to ensure optimal activation [35].

Taken together, CMA plays an essential role in physiological processes, including proteostasis, metabolic pathways, cellular energetics, the cell cycle, cell stemness, and immune responses. CMA activity is decreased along with aging, which can be attributed to the lower stability of LAMP2A at the lysosomal membrane conferred by alterations of lipid composition [8]. Although there is cross-talk between macroautophagy and CMA to compensate for the functional impairment of each [36], their functional redundancy is not sufficient for complete recovery. Both CMA and macroautophagy functionally decline during aging, suggesting that CMA malfunction may play a pivotal role in disease pathogenesis, particularly associated with aging.

## Pathogenic implication of CMA

CMA malfunction has been widely implicated in aging-associated pathologies, including neurodegenerative disorders, metabolic disorders, and cancers [37]. Reduced CMA is implicated in Parkinson’s disease (PD) pathogenesis through the inability to degrade mutant alpha-synuclein [38]. There are several CMA substrates linked to PD development, including PARK7, leucine-rich repeat serine/threonine-protein kinase 2 (LRRK2), and ubiquitin carboxyl-terminal hydrolase isozyme L1 (UCHL1) [8]. CMA malfunction has also been implicated in the pathogenesis of other neurodegenerative disorders, including Alzheimer’s disease, frontotemporal lobar degeneration, amyotrophic lateral sclerosis, and Huntington’s disease [8]. Impaired degradation of CMA substrates is caused by pathogenic variants, which are targeted to lysosomes but fail to degrade and can inhibit CMA, resulting in the accumulation of toxic aggregates. Pathogenic variants may also diminish CMA activity by affecting lysosome biogenesis; thus, the accumulation of variant proteins at the lysosomal surface may further disrupt CMA-mediated proteostasis [8]. Reduced LAMP2A protein levels have been demonstrated in both familial and idiopathic PD patients’ brains [39], suggesting the existence of a variety of CMA substrates and mechanisms for CMA impairment associated with the development of neurodegenerative disorders.

Because CMA has a pivotal role in glucose and lipid metabolism, the involvement of CMA malfunction in metabolic disorders has been reported [40]. The potential involvement of reduced CMA has been demonstrated in both non-alcoholic fatty liver disease and alcoholic liver disease [41, 42]. Experimental blockage of CMA induces metabolic dysregulation in the liver, resulting in hepatic glycogen depletion and hepatosteatosis [43]. CMA is suppressed in the renal cortex during acute diabetes mellitus, resulting in the accumulation of proteins with KFERQ motifs, including paired box 2 (PAX2) and glyceraldehyde 3-phosphate dehydrogenase (GAPDH). PAX2 is a transcription factor that regulates epithelial cell differentiation of the fetal kidney and ureter [40]. Thus, the accumulation of specific proteins by impaired CMA may be causally associated with the development of diabetic-induced renal hypertrophy [44]. Mucolipidosis type IV (MLIV) is a lysosomal storage disorder caused by mutations of transient receptor potential mucolipin-1 (TRPML1), which is a member of the TRP cation channel gene family and localized to lysosomes [45]. CMA is defective in fibroblasts isolated from MLIV patients. Increased levels of oxidized proteins in MLIV fibroblasts compared to control fibroblasts implicate deficient CMA in the pathogenesis of MLIV development. Protein interactions between TRPML1 and HSC70 as well as TRPML1 and HSP40 suggest that TRPML1 may have a regulatory role in CMA activity and may explain the mechanism for deficient CMA in MLIV [45].

It has been reported that CMA activity is upregulated in most cancer cell lines and increased LAMP2A expression levels are observed in a wide array of human tumors [8]. CMA inhibition suppresses cell survival and tumorigenicity, indicating that CMA activation is involved in the mechanisms of tumor progression. The survival benefit for cancer cells can be at least partly attributed to CMA-mediated degradation of damaged proteins for maintaining proteostasis, resulting in enhanced resistance to oxidative stress and DNA damage [8]. It has been reported that CMA prevents apoptosis and contribute to resistance to oxaliplatin in hepatocellular carcinoma by degrading the apoptosis trigger cyclin D1 [46] and to irradiation by degrading HMGB1 [47]. Degradation of the acetyltransferase p300/CBP by CMA confers resistance to 5-fluorouracil in colorectal cancer [48]. CMA-mediated degradation of glycolytic enzymes is linked to the Warburg effect, which is the metabolic shift to glycolysis, an essential energy source for tumor growth and proliferation [49]. Accordingly, it is likely that inhibition of CMA has anticancer effects by increasing chemosensitivity via enhanced damage-induced cell death. Actually, we have recently reported that CMA is involved in the chemoresistance in lung cancer cell lines [9]. In line with macroautophagy, it is important to note that CMA has an anti-oncogenic role in non-transformed cells in physiological condition. CMA is involved in the mechanisms for DNA repair and may prevent malignant transformation by maintaining genome stability [8]. CMA may prevent cellular transformation by accelerating the proteasomal degradation of MYC [50], indicating that aging-associated functional loss of CMA may contribute to malignant transformation. Hence, the anti- and pro-oncogenic effects of CMA are probably numerous and depend on the types and stages of cancer development [8, 51].

Although potential participation of CMA in the pathogeneses of a variety of disorders, including heart, liver, and kidney diseases has been demonstrated, among pulmonary diseases, only lung cancer and COPD have been reported to be associated with CMA malfunction [8].

## CMA in lung cancer pathogenesis

Upregulation of the CMA pathway is associated with positive modulation of cancer cell survival and growth [49]. However, CMA in non-tumorigenic cells has an anti-tumor functions, preventing malignant transformation [50]. COPD is recognized to be a major risk factor for lung cancer development, and both macroautophagy and CMA may have inhibitory role in malignant transformation [52]. Accordingly, reduced autophagy can be a part of the mechanisms for higher frequency of lung cancer development in COPD patients. However, the causal link between CMA and lung cancer development remains to be established. In contrast, CMA becomes highly active to sustain important pro-oncogenic functions after malignant transformation. It has been reported that LAMP2A expression, a surrogate for CMA activity in human tumors, is elevated in many human tumors, including gastric cancer, colon cancer, breast cancer, and non-small cell lung cancer (NSCLC) [53]. Indeed, CMA-induced degradation of misfolded nuclear receptor corepressor (NCOR) proteins has an important role in the neutralization of ER stress in NSCLC [54]. CMA-mediated stabilization of MCL1, a pro-survival protein stabilization, has been demonstrated to contribute to survival in NSCLC cell lines [55]. Furthermore, CMA-mediated degradation of damaged proteins may confer resistance to chemotherapeutic agents-mediated oxidative stress and DNA damage [8].

**Fig. 3:**
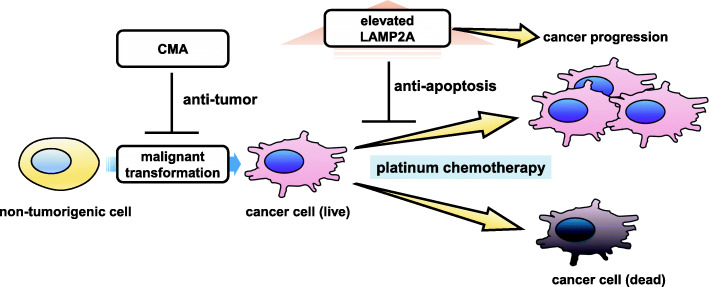
Elevated LAMP2A in chemoresistance in NSCLC. Elevated LAMP2A is associated with resistance to platinum-based chemotherapy through anti-apoptotic property of CMA, resulting in cancer progression

We examined whether CMA modulates the response to platinum-based chemotherapy by regulating apoptotic signaling in NSCLC. In line with previous findings, immunohistochemical analysis revealed significantly higher LAMP2A expression levels in NSCLC compared to normal lung samples and high expression levels of LAMP2A were significantly associated with poor relapse free survival [9]. Furthermore, LAMP2A expression correlates with responses of NSCLC patients to platinum-based chemotherapy. In in vitro experiments using NSCLC cell lines, CMA blockage suppresses cell proliferation and increases sensitivity to chemotherapeutic drugs through enhancing intrinsic apoptosis signaling [9]. In vivo cancer xenograft models using NSCLC cell lines with LAMP2A knockdown show reduced tumorigenic ability and increased sensitivity to cisplatin treatment [9]. Understanding the precise function of CMA in NSCLC may allow for the use of LAMP2A as a biomarker for predicting patient response to platinum-based chemotherapy (Fig. 3) and further assist in the development of new therapeutic strategies against chemoresistant NSCLC.

## CMA in COPD pathogenesis

**Fig. 4:**
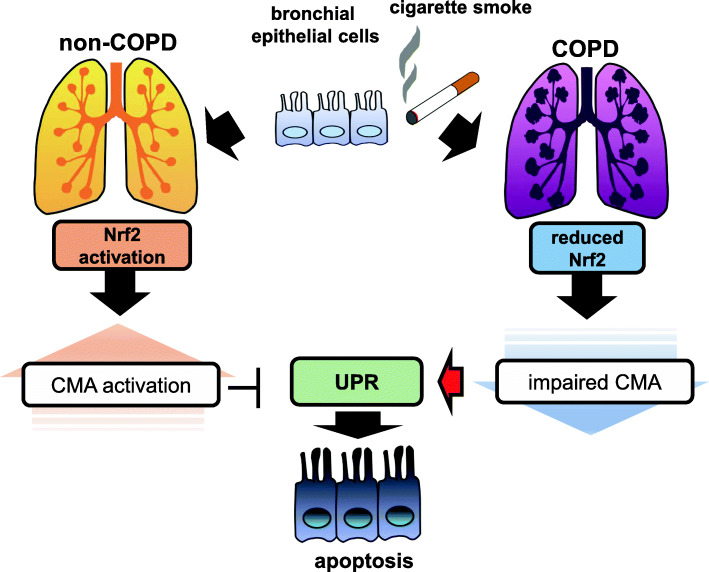
Role of CMA in COPD pathogenesis. CMA activity is enhanced through Nrf2-mediated LAMP2A expression in response to cigarette smoke exposure. CMA prevent apoptosis by attenuating unfolded protein response (UPR) in non-COPD lung. Impaired CMA activity resulting from reduced Nrf2 expression leads to enhanced UPR with concomitantly enhanced apoptosis during COPD pathogenesis

COPD, which is mainly caused by CS exposure, is a leading cause of death worldwide [56]. COPD is a representative aging-associated pulmonary disease characterized by progressive airflow limitation, which is progressive even after smoking cessation and aggressive medical intervention. Accumulating evidence indicates that oxidative stress may have an essential role in COPD development [57–59]. CS induces oxidative modifications to a variety of lung macromolecules including proteins, resulting in the accumulation of damaged and misfolded proteins with a concomitantly enhanced unfolded protein response (UPR) [60, 61]. UPR is generally a cytoprotective mechanism, but also induces apoptosis during excessive ER stress [62]. A recent paper shows that UPR actives CMA via p38-mediated phosphorylation of LAMP2A, indicating that CMA-regulated proteostasis may have an essential role in COPD pathogenesis with increased UPR [63]. In addition, an inhibitory role for CMA in CS extract (CSE)-induced epithelial cell apoptosis has been reported for immortalized BEAS-2B bronchial epithelial cells [64]. Our in vitro experiments have shown that CSE induces CMA activation of LAMP2A expression through Nrf2-regulated transactivation [13]. CMA inhibition enhances the UPR, accompanied by increased apoptosis in response to CSE exposure, which is clearly reversed by LAMP2A overexpression in human bronchial epithelial cells (HBECs), indicating functional crosstalk between UPR and CMA during CSE exposure [13]. Among UPR proteins, CHOP expression is responsible for CS-induced and CMA-regulated apoptosis in HBECs. Compared with never -smokers and non-COPD smokers, reduced Nrf2 and LAMP2A expression levels have been demonstrated in airway epithelial cells in COPD lungs by immunohistochemical evaluation. Both Nrf2 and LAMP2A expression levels are significantly reduced in HBECs isolated from COPD patients and there is a positive correlation between Nrf2 and LAMP2A expression levels are detected. LAMP2A expression levels in HBECs are significantly correlated with pulmonary function tests, indicating that impaired CMA modulated by Nrf2 may be causally associated with COPD development through enhanced UPR-mediated apoptosis in lung epithelial cells (Fig. 4). Accordingly, it is plausible that activating CMA can be a an antiapoptotic modality for COPD treatment [13].

There is compensatory functional crosstalk between macroautophagy and CMA, and increased CMA activation has been demonstrated in macroautophagy-deficient cells [36]. Accordingly, we have observed that CMA inhibition by LAMP2A knockdown enhances macroautophagy, and macroautophagy inhibition by ATG5 knockdown enhances CMA, in response to CSE exposure in HBECs, indicating the existence of compensatory mechanisms between them (unpublished data). However, the compensatory interplay between these proteostatic machineries are not totally redundant, and since CMA is responsible for selective degradation of proteins with the KFERQ peptide motif [8], insufficient CMA cannot be completely compensated for by upregulation of macroautophagy. Additionally, although macroautophagy activation reaches its maximal peak shortly after stimulation, CMA activation is gradual and persists at maximal activity for several days [37]. Indeed, macroautophagy activity peaks at 24 h after CSE treatment, but LAMP2A expression levels peak at 48 h and are maintained at 72 h according to our experiments, suggesting that CMA activity can be a more critical determinant for cell fate, especially in the later phases following CSE exposure [3, 13]. The activity and role of macroautophagy in COPD pathogenesis remains controversial with respect to differences in pathogenic cell fate between cellular senescence and programmed cell death (PCD) [65]. Although insufficient macroautophagy/mitophagy is responsible for the progression of cellular senescence [3, 5], excessive macroautophagy/ mitophagy may induce PCD in lung epithelial cells [11]. Therefore, the role of functional crosstalk between macroautophagy and CMA in determining cell fate is still uncertain during COPD pathogenesis. However, in line with previous findings [27], we have observed exaggerated UPR and apoptosis in the case of simultaneous ATG5 and LAMP2A knockdown (unpublished data), indicating that both macroautophagy and CMA activity have cell protective properties during CSE exposure and that reduced CMA may have a more pronounced role in COPD pathogenesis via excessive apoptosis in the setting of insufficient macroautophagy. Furthermore, CMA has also been postulated to be responsible for regulating cellular senescence in response to oxidative stress through a variety of mechanism [66]. We speculate that both macroautophagy and CMA have key regulatory roles in COPD pathogenesis through preventing not only PCD but also cellular senescence.

We have recently reported the involvement of increased ferroptosis, a form of regulated necrosis associated with lipid peroxidation, in COPD pathogenesis [67]. Intriguingly, CMA may have an inconsistent role in regulating ferroptosis. CMA may activate ferroptosis via glutathione peroxidase 4 (GPX4) degradation but conversely may also prevent ferroptosis by increasing glutathione (GSH) levels [68, 69]. Thus, the precise role of CMA in regulating ferroptosis with respect to COPD pathogenesis remains uncertain.

## Conclusions

Recent research advances have shed light on both the molecular mechanisms and physiological and pathological roles of CMA. A growing body of evidence implicates CMA in the pathogenesis of a wide array of diseases in multiple organs, and CMA regulation is a potential therapeutic target. Although there is no established agent for modifying CMA activity for clinical application, recent study has shown the potential therapeutic efficacy of specific CMA activation by retinoic acid receptor alpha (RARα) antagonists for mouse model of Parkinson disease [70]. Accordingly, CMA activation by using these antagonists should be studied as a potential therapeutic approach for COPD. With respect to pulmonary disorders, the involvement of CMA has been demonstrated only in lung cancer and COPD pathogenesis through modulating apoptosis. Due to the canonical role of CMA in cell physiology, its participation should be examined in other common pulmonary disorders, including interstitial pneumonia, bronchial asthma, and lung infectious diseases in terms of regulating proteostasis, metabolic pathways, and immune responses.

## Data Availability

Not applicable

## Abbreviations

ATG5: Autophagy related 5
CBP: Creb-binding protein
CDK: Cyclin-dependent kinase
CSE: Cigarette smoke extract
cGAS: Cyclic GMP-AMP synthase
CHIP: Carboxyl terminus of HSC70-interacting protein
CHK1: Checkpoint kinase 1
CMA: Chaperone-mediated autophagy
COPD: Chronic obstructive pulmonary disease
ER: Endoplasmic reticulum
GAPDH: Glyceraldehyde 3-phosphate dehydrogenase
GPX4: Glutathione peroxidase 4
HBEC: Human bronchial epithelial cell
HIF-1α: Hypoxia-inducible factor-1 subunit α
HIP: HSP70-interacting protein
HMGB1: High mobility group box-1 protein
HOP: HSP70-HSP90 organizing protein
HSC: Hematopoietic stem-cell
HSC70: Heat shock cognate 71 kDa protein
HSP40: Heat shock protein 40
LAMP2A: Lysosomal-associated membrane protein type 2A
LRRK2: Leucine-rich repeat serine/threonine-protein kinase 2
MCL1: Myeloid cell leukemia sequence 1
MLIV: Mucolipidosis type IV
NCOR: Nuclear receptor corepressor
NFAT1: Nuclear factor of activated T cells 1
NF-κB: Nuclear factor-κB
Nrf2: Nuclear factor-erythroid 2-related factor 2
NSCLC: Non-small cell lung cancer
PARK7: Parkinson disease protein 7
PAX2: Paired box 2
PCD: Programmed cell death
PD: Parkinson’s disease
PHLPP1: PH domain and leucine rich repeat protein phosphatase 1
PPARα: Peroxisome proliferator-activated receptor-alpha
STING: Stimulator of interferon genes
TORC2: Target of rapamycin complex-2
TRPML1: Transient receptor potential mucolipin-1
UPR: Unfolded protein response
